# Study of diffusion‐weighted magnetic resonance imaging in the evaluation of the response to AAV2‐VEGF‐Trap neoadjuvant treatment in a triple‐negative breast cancer animal model

**DOI:** 10.1002/cam4.1963

**Published:** 2019-03-21

**Authors:** Jianhua Li, Pengjin Zhu, Lei Wang, Li Yang, Liqun Zou, Fabao Gao

**Affiliations:** ^1^ The First Department of Oncology West China Hospital, Sichuan University Chengdu China; ^2^ Department of Oncology Linfen Central Hospital Linfen China; ^3^ Department of Radiology West China Hospital, Sichuan University Chengdu China; ^4^ Department of State/National Key Laboratory of Biotherapy Sichuan University Chengdu China

**Keywords:** AAV2‐VEGF‐Trap, angiogenesis, apparent diffusion coefficient, diffusion‐weighted magnetic resonance imaging, in vivo fluorescence imaging, triple‐negative breast cancer, Δ ADC

## Abstract

**Objective:**

Evaluation of the efficacy of adeno‐associated virus 2 mediated gene transfer of vascular endothelial growth factor Trap (AAV2‐VEGF‐Trap) alone or combination with paclitaxel in a mouse model of triple‐negative breast cancer (TNBC) using diffusion‐weighted magnetic resonance imaging (DW‐MRI) and in vivo fluorescence imaging.

**Materials and Methods:**

Xenografted TNBC tumors were established by subcutaneous injection of MDA‐MB‐231 cells into nude mice. Tumors were treated with AAV2‐VEGF‐Trap, paclitaxel, AAV2‐VEGF‐Trap combined with paclitaxel and control. A 7.0‐Tesla magnetic resonance (MR) was used to obtain the apparent diffusion coefficient (ADC) values and ΔADC values. In vivo fluorescence imaging coupled with the optical imaging probe AngioSense680 EX was acquired to obtain average luminous intensity values. Immunohistochemical staining of tumor Ki‐67 and vascular endothelial cell marker antigen (CD31) were used to evaluate the effects on tumor proliferation and angiogenesis.

**Results:**

The combination of AAV2‐VEGF‐Trap with paclitaxel exhibited greater tumor growth inhibition compared with the other groups. The ADC values in the paclitaxel group and the AAV2‐VEGF‐Trap in combination with paclitaxel group were significant greater compared with the control group, and the ΔADC values of all treatment groups were significantly increased compared with the control group on the 14th day after administration. Decreased microvessel density and luminous intensity in the treatment groups that contain AAV2‐VEGF‐Trap were observed. Reduced proliferation activity was noted in groups that contained paclitaxel.

**Conclusion:**

AAV2‐VEGF‐Trap inhibits TNBC growth though inhibiting tumor neovascularization with a single intravenous injection, and AAV2‐VEGF‐Trap exhibits a synergistic effect when used in combination with paclitaxel for TNBC neoadjuvant therapy. In vivo fluorescence imaging can detect the anti‐angiogenesis effect of AAV2‐VEGF‐Trap early and noninvasively. DW‐MRI can longitudinally monitor the neoadjuvant efficacy of TNBC.

## 1 INTRODUCTION

Triple‐negative breast cancers (TNBC) account for 15%‐20% of breast cancer, commonly arising in African and African‐American premenopausal women.^1^ TNBC patients cannot benefit from endocrine therapy and anti‐Her2 targeted therapy given the lack of hormone receptor and ERBB2 receptor expression.^2^ Therefore, the conventional anti‐cancer approach (surgery, chemotherapy, and radiotherapy) remains the major treatment option for TNBC.^3^ Some local advanced patients can also benefit from neoadjuvant chemotherapy.^4^

Angiogenesis is mainly regulated by vascular endothelial growth factor (VEGF)/VEGF receptor family and is closely related to growth and metastasis in solid tumor.^5^ Studies have demonstrated that high VEGF expression in TNBC is associated with poor prognosis,^6^ suggesting that patients with TNBC might benefit from anti‐angiogenesis therapy. The highest affinity VEGF blocker, VEGF‐Trap, was approved for the treatment of metastatic colon cancer by the FDA in 2012.^7^ A recent study revealed increased efficacy for the addition of VEGF‐Trap in neoadjuvant/adjuvant chemotherapy for TNBC.^8^ However, due to its short half‐life, VEGF‐Trap requires long‐term and repeated injections, which are accompanied by a high cost and patient resistance. Gene therapy using adeno‐associated virus 2 (AAV2) to deliver VEGF‐Trap achieved long‐term expression of VEGF‐Trap in vitro and in vivo.^9^ Animal studies have demonstrated that the growth of breast cancer and glioma can be inhibited by a single injection of AAV2‐VEGF‐Trap.^9^, ^10^ In this report, we aimed to evaluate the antitumor efficacy of AAV2‐VEGF‐Trap alone or combination with paclitaxel in a TNBC animal model.

Given that anti‐angiogenesis therapies have become a widely accepted approach for tumor therapy, researchers have also focused on how to evaluate the efficacy of anti‐angiogenic agents. Micro vascular density (MVD) obtained via IHC staining of CD31 on tumor biopsies is an accuracy biomarker for assessing the efficacy of anti‐angiogenic agents.^11^ However, invasive and impractical multiple samplings limited its application. In vivo fluorescence imaging has been widely used to measure tumor angiogenesis in mice Xenografts.^12^ AngioSense680 EX is a near‐infrared‐labeled fluorescent macromolecule that exhibits inherent advantages for imaging of blood vessels as it localizes in the vasculature for extended periods of time.^13^ Previous studies demonstrated the best time point for quantitative measurement of angiogenesis and blood vessel density was 24 hours after tail vein injection of AngioSense680 EX.^14^, ^15^ In the present study, we utilize in vivo fluorescence imaging coupled with imaging probe AngioSense680 EX to characterize the inhibitory potential of AAV2‐VEGF‐Trap in angiogenesis.

Diffusion‐weighted magnetic resonance imaging (DW‐MRI) is a well‐established technique to diagnose breast cancer and to identify cancer metastasis.^16^ The apparent diffusion coefficient (ADC) value quantified by DW‐MRI, which provides a measurement the Brownian motion of water, is negatively correlated with tumor cell density.^17^ Studies demonstrated that the ADC value increases after successful chemo‐/radiotherapy in various cancers, including malignant glioma, prostate cancer, and breast cancer.^18^, ^19^, ^20^ Effective treatment results in necrosis or apoptosis of tumor cells and a decrease in tumor cell density; theoretically, the ADC value is increased. However, another study reported that anti‐VEGF therapies reduced ADC values in the intracranial metastasis model of breast cancer due to the reduction in tissue perfusion.^21^ Remarkably, for patients with abdominal metastases tumor, the ADC values increased significantly after treatment with VEGF inhibitors.^22^ Therefore, the variation in ADC values after successful anti‐angiogenesis treatment of TNBC remains unclear. In the present study, we used DW‐MRI to assess the anti‐TNBC efficacy of AAV2‐VEGF‐Trap alone or in combination with paclitaxel.

## 2 MATERIALS AND METHODS

### 2.1 Cell line and reagents

MDA‐MB‐231 cell lines were purchased from the ATCC (American Type Culture Collection, Manassas, VA, USA) and stored according to supplier's instructions. AAV2‐VEGF‐Trap was constructed in the State Key Laboratory of Biotherapy, West China Hospital of Sichuan University. Paclitaxel was obtained from Beijing Ruikang Pharmaceutical Industry (China). AngioSense680 EX was obtained from PerkinElmer, Inc (Boston, MA, USA). DMEM and fetal bovine serum were purchased from Gibco (USA). Anti‐Ki‐67 and anti‐CD31 are rabbit polyclonal antibodies purchased from Abcam (Shanghai, China).

MDA‐MB‐231 cells were cultured in DMEM supplemented with 10% fetal bovine serum and antibiotics (100 IU/mL penicillin and 100 µg/mL streptomycin) at 37°C in a humidified 5% CO_2_ atmosphere.

### 2.2 Breast carcinoma model

**Figure 1:**
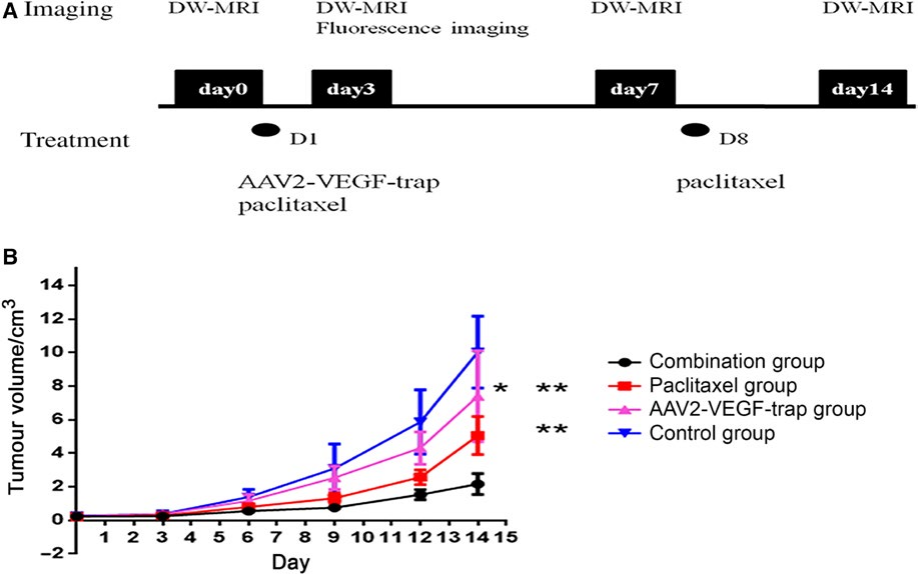
AAV2‐VEGF‐Trap exhibits synergistic effects with paclitaxel in TNBC xenograft models. Mice bearing ~220 mm^3^ primary breast tumors were randomized and administered drug as follows: control group (normal saline intraperitoneal injection once a week for a total of two times), AAV2‐VEGF‐Trap group (2 × 10^9 ^vg via one tail vein injection), paclitaxel group (30 mg/kg intraperitoneal injection once a week for a total of two times), combination group (AAV2‐VEGF‐Trap combined with paclitaxel dose and use are the same as described for monotherapy). A, Schedule for the different imaging technologies performed and treatments administered. B, Tumor growth curves of four groups are presented (*P < 0.05 AAV2‐VEGF‐Trap group compared with control group on the 14th day of administration; **P < 0.01 AAV2‐VEGF‐Trap group or paclitaxel group compared with combination group on the 14th day of administration)

All the animal studies were approved by the ethics committee of West China Hospital of Sichuan University and complied with the regulation on the administration of experimental animals.Female nude mice (6‐8 weeks of age) were purchased from Chengdu Dashuo Biological Technology Co., Ltd, Chengdu, China. All animals were housed under pathogen‐free conditions and fed autoclaved pellets and water. Mice were inoculated with 1 × 10^6^ MDA‐MB‐231 tumor cells. After mice developed visible tumors, the tumor‐bearing mice were randomly divided into four groups. Mice in the AAV2‐VEGF‐Trap group were administered 2 × 10^9^ vg AAV2‐VEGF‐Trap (4 μL in 50 μL PBS) through tail vein injection. Mice in the paclitaxel group were administered 30 mg/kg paclitaxel dissolved in 0.2 mL normal saline via an intra peritoneal injection once a week for a total of two times. Mice in the combination group (AAV2‐VEGF‐Trap combined with paclitaxel) were administered 2 × 10^9^ vg AAV2‐VEGF‐Trap (4 μL in 50 μL PBS) through tail vein injection and 30 mg/kg paclitaxel dissolved in 0.2 mL normal saline via intra peritoneal injection once a week for a total of two times. Tumor size was measured before treatment and on the 3rd, 7th, and 14th day after the administration using vernier calipers, and tumor volume calculated as 0.5 × long diameter × short diameter.^2^ In vivo fluorescence imaging was performed on the 3rd day after administration. MR‐DWI examination was performed before treatment (day 0) and on the 3rd, 7th, and 14th day after administration followed by measurement of ADC/ΔADC values. Figure 1A indicates when different imaging technologies were performed and when treatments were administered.

### 2.3 In vivo fluorescence imaging

Nude mice were anesthetized using 1‐3% isoflurane gas and scanned using the IVIS Spectrum System (Xenogen, Alameda, CA, USA) on the third day after treatment. AngioSense680 EX (2 mg in 0.1 mL PBS) was administered though tail vein injection 24 hours before the imaging scan. ROI was manually sketched on the tumor luminescence area of each nude mouse. The average luminescence intensity was automatically generated using Spectrum Living Image 4.0 optical software (Xenogen).

### 2.4 MR‐DWI imaging

The MR scan was performed in four groups before treatment (day 0) and on day 3, day 7, and day 14 after the administration. All MR images were obtained with a 7.0 Tesla MR scanner (Bruker BioSpec 70/30, Ettlingen, Germany). Multislice multiecho (MSME) T1‐weighted images were obtained using the following parameters: field of view (FOV) = 30 × 30 mm, repetition time (TR) = 500 ms, echo time (TE) = 8.3 ms, number of excitations (NEX) = 2, matrix size = 256 × 256, Flip angle (FA) = 180°, slice thickness = 1 mm, slice gap = 0 mm, and acquisition time = 2 minutes 18 seconds. Turbo Rapid Acquisition Relaxation Enhancement (RARE) T2‐weighted images were obtained using the following parameters: FOV = 33 × 33 mm, TR = 2500 ms, TE = 33 ms, NEX = 2, matrix size = 256 × 256, FA = 180°, slice thickness = 1 mm, slice gap = 0 mm, and acquisition time = 2 minutes 55 seconds. Diffusion‐weighted images (DWIs) were collected using the following sequence parameters: FOV = 33 × 33 mm, TR = 3000 ms, TE = 45 ms, matrix size = 256 × 256, slice thickness = 1 mm, slice gap = 0 mm, and *b* values = 0 and 1000 s/mm^2^. Apparent diffusion coefficient (ADC) maps were generated automatically using software, and then regions of interest (ROIs) were drawn in the central tumor bypassing necrotic or cystic areas by two experienced radiologists who did not know the grouping information. The ADC values of 5 ROIs in each mouse were measured, and the mean value was recorded. ΔADC value is calculated as follows: (Average ADC value of each time point after treatment‐average ADC value before treatment)/(average ADC value before treatment).

### 2.5 Immunohistochemistry

After completion of the MR scan on day 14, the nude mice were sacrificed. Tumor tissues were fixed in 4% paraformaldehyde, embedded in paraffin and then cut into sections. The tumor tissue sections were stained for blood vessels using an anti‐CD31 antibody and for proliferative cells using an anti‐Ki‐67 antibody.

Micro vessel density (MVD) was evaluated according to a previously described method.^23^ Slides were scanned at low magnification (40 ~ 100 times) to identify the area with the highest density of blood vessels called "hot spots" and then scored on HPFs (x 200). Similarly, five different fields were selected randomly, and 100 tumor cells were counted at 400×. The percentage of Ki‐67‐positive cells was calculated.

### 2.6 Statistical analysis

SPSS 16.0 (IBM Corporation, Armonk, New York, USA) was used for statistical analysis. All quantitative data were expressed as the mean ± SD. One‐way analysis of variance and Student's *t* test were used for comparisons among multiple groups and between two groups, respectively. *P*‐values <0.05 were considered statistically significant. The correlation analysis was performed using Pearson correlation analysis, and the correlation relationship was represented by the correlation coefficient *R* ^2^.

## 3 RESULTS

### 3.1 AAV2‐VEGF‐Trap enhanced the antitumor efficacy of paclitaxel in TNBC xenograft Models

To ascertain the effects of AAV2‐VEGF‐Trap against TNBC, mice with established primary MDA‐MB‐231 tumors approximately 220 mm^3^ in volume were randomized and treated for 2 weeks with control, AAV2‐VEGF‐Trap, paclitaxel, or AAV2‐VEGF‐Trap in combination with paclitaxel. Response to therapy was assessed by primary tumor burden, tumor angiogenesis, and tumor proliferation.

First, we assessed residual primary tumor volume. Compared with the control group, the AAV2‐VEGF‐Trap group significantly reduced the terminal tumor mass (*P* = 0.03; Figure 1). Compared with the paclitaxel or AAV2‐VEGF‐Trap group, the combination group exhibited a significant further reduction in terminal tumor mass (*P* < 0.0001 and *P* = 0.0002, respectively; Figure 1). These results indicated that AAV2‐VEGF‐Trap effectively inhibits the growth of TNBC alone or combined with paclitaxel.

**Figure 2:**
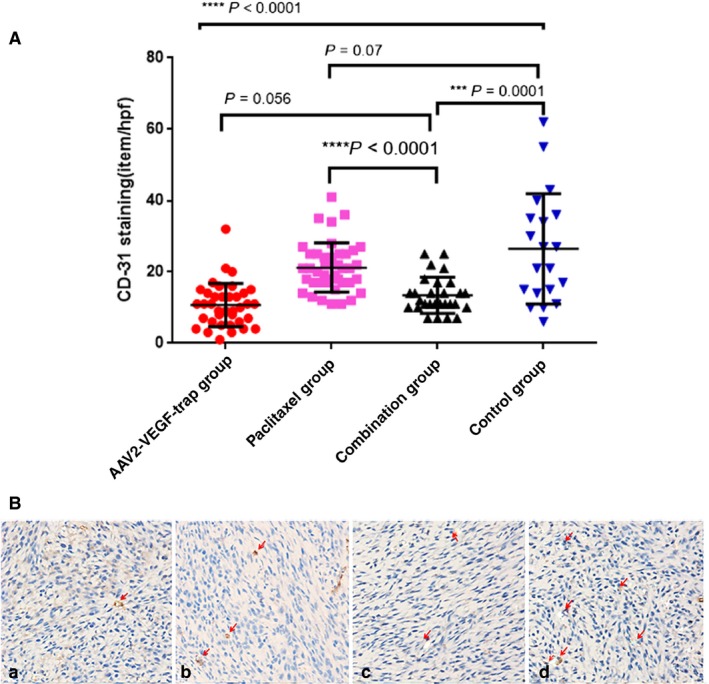
AAV2‐VEGF‐Trap exhibits anti‐angiogenesis effects in TNBC xenograft models. After two weeks of treatment, the nude mice were euthanized, and tumor tissues were collected for CD31 immunohistochemical staining. (A) The number of CD31‐positive cells in the four groups. Values are expressed as the mean ± SD. (B) A, B, C, and D were representative CD31 (x 200) immunohistochemical images of the combination group, paclitaxel group, AAV2‐VEGF‐Trap group, and control group, respectively

Next, tumor neovascularization via immunohistochemistry staining of CD31 was evaluated. Compared with the control group, AAV2‐VEGF‐Trap monotherapy and combination therapy significantly inhibited tumor angiogenesis (*P* < 0.0001 and *P* = 0.001, respectively; Figure 2A). However, the difference between the paclitaxel group and control group was not statistically significant (*P* = 0.07; Figure 2A). These results demonstrate that the MVD values of groups containing AAV2‐VEGF‐Trap were significantly reduced compared with non‐AAV2‐VEGF‐Trap treatment groups.

**Figure 3:**
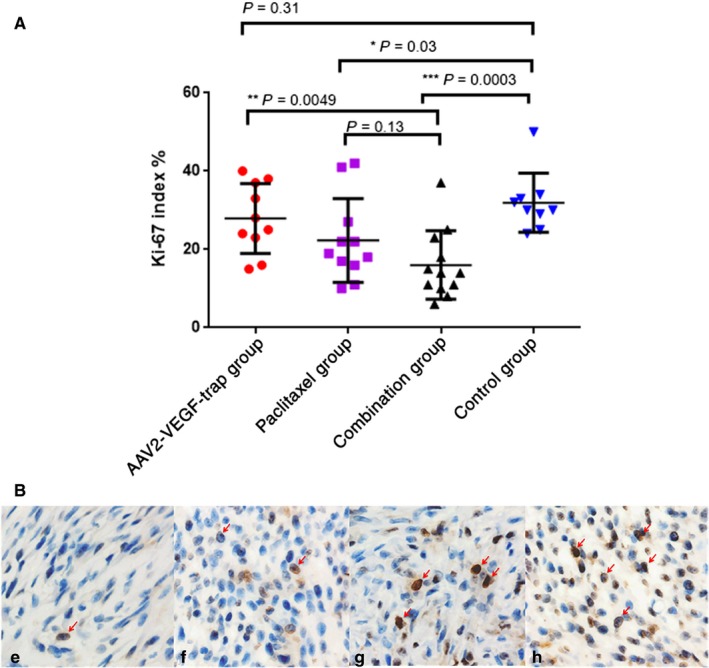
Differential treatment effects on tumor proliferation. After 2 wk of treatment, the nude mice were euthanized, and tumor tissues were collected for Ki‐67 immunohistochemical staining. (A) The percentage of Ki‐67‐positive cells in the four groups, and values are expressed as the mean ± SD. (B) E, F, G, and H are representative Ki‐67 (×400) immunohistochemical images of the combination group, paclitaxel group, AAV2‐VEGF‐Trap group, and control group, respectively. Cells with nuclear staining are noted as actively proliferating tumor cells

As a nuclear antigen, Ki‐67 is expressed in the G1, S, and G2 phases of the cell cycle and is an established proliferation marker in breast cancer.^24^ A recent study demonstrated that a decrease in the Ki‐67 index after treatment indicates effective neoadjuvant chemotherapy and better prognosis.^25^ Compared with the control group, the Ki‐67 index in the paclitaxel group and combination group was significantly decreased (*P* = 0.03 and *P* = 0.0003, respectively; Figure 3A), whereas no significant reduction was noted in the AAV2‐VEGF‐Trap group (*P* = 0.31).

Based on tumor burden, MVD value, and Ki‐67 index results after treatment, it can be concluded that AAV2‐VEGF‐Trap inhibits TNBC growth though inhibiting tumor neovascularization after a single intravenous injection. Moreover, AAV2‐VEGF‐Trap in combination with paclitaxel achieves improved efficacy in suppressing tumor growth, suggesting that this approach can be a promising neoadjuvant therapy option for TNBC.

### 3.2 In vivo fluorescence imaging can assess the anti‐angiogenesis effect of AAV2‐VEGF‐Trap

**Figure 4:**
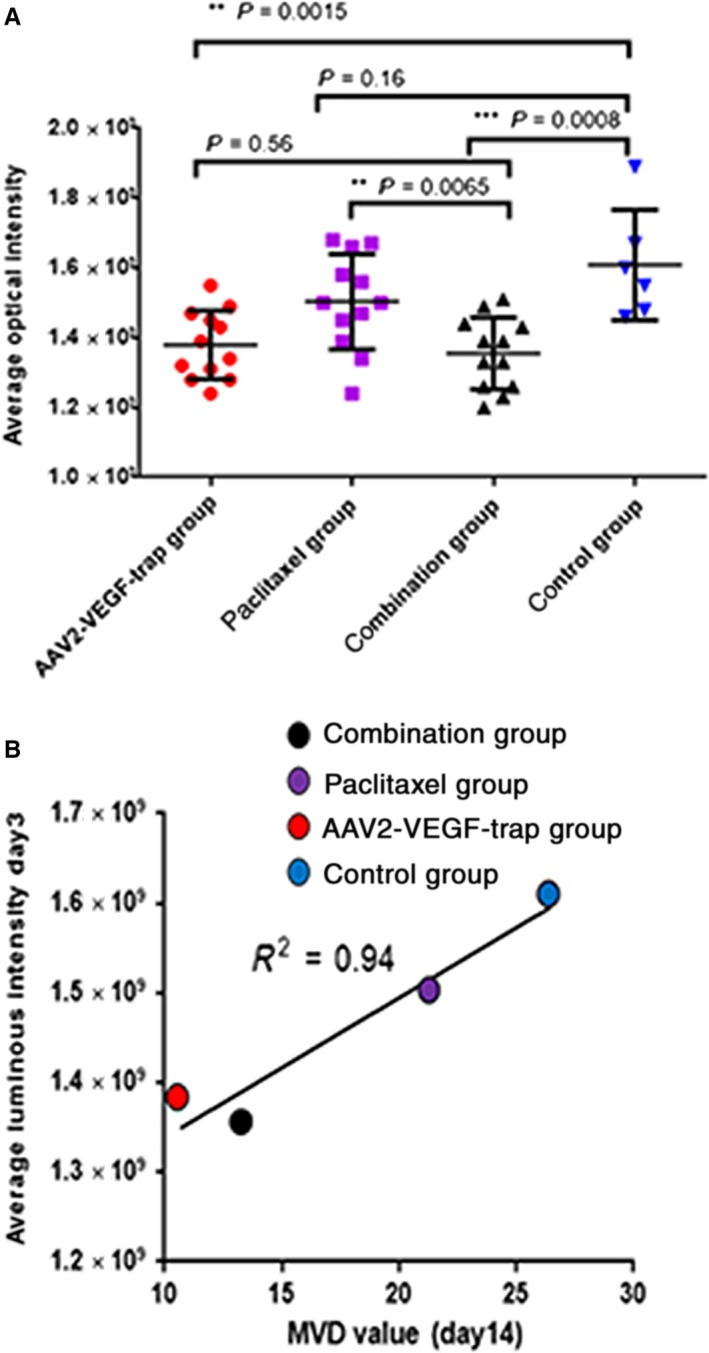
In vivo fluorescence imaging evaluates the anti‐angiogenic efficacy of AAV2‐VEGF‐Trap. Tumor‐bearing mice were imaged on the 3rd day after dosing initiation. At 24 h before the imaging scan, mice were injected with AngioSense680 EX though the tail vein. A, Average luminous intensity of four groups; values are expressed as the mean ± SD. B, Correlation analysis between average luminous intensity and MVD value

We tracked tumor angiogenesis on the 3rd day after treatment by in vivo fluorescence imaging coupled with AngioSense680 EX, which is a specific in vivo blood pool vascular fluorescent imaging probe. The average luminous intensity of the AAV2‐VEGF‐Trap group and the AAV2‐VEGF‐Trap in combination with paclitaxel group were significantly decreased compared with the control group (*P* = 0.0015 and *P* = 0.0008, respectively; Figure 4B). The difference between the paclitaxel group and the control group was not statistically significant (*P* = 0.16; Figure 4B). These results indicate that the mean luminous intensity of both groups containing AAV2‐VEGF‐Trap was significantly reduced compared with the groups without AAV2‐VEGF‐Trap (Figure 4B). Moreover, average luminous intensity was positively correlated with MVD (*R* ^2 ^= 0.94, Figure 4C).

### 3.3 MR‐DWI can evaluate the efficacy of neoadjuvant therapy of TNBC

**Figure 5:**
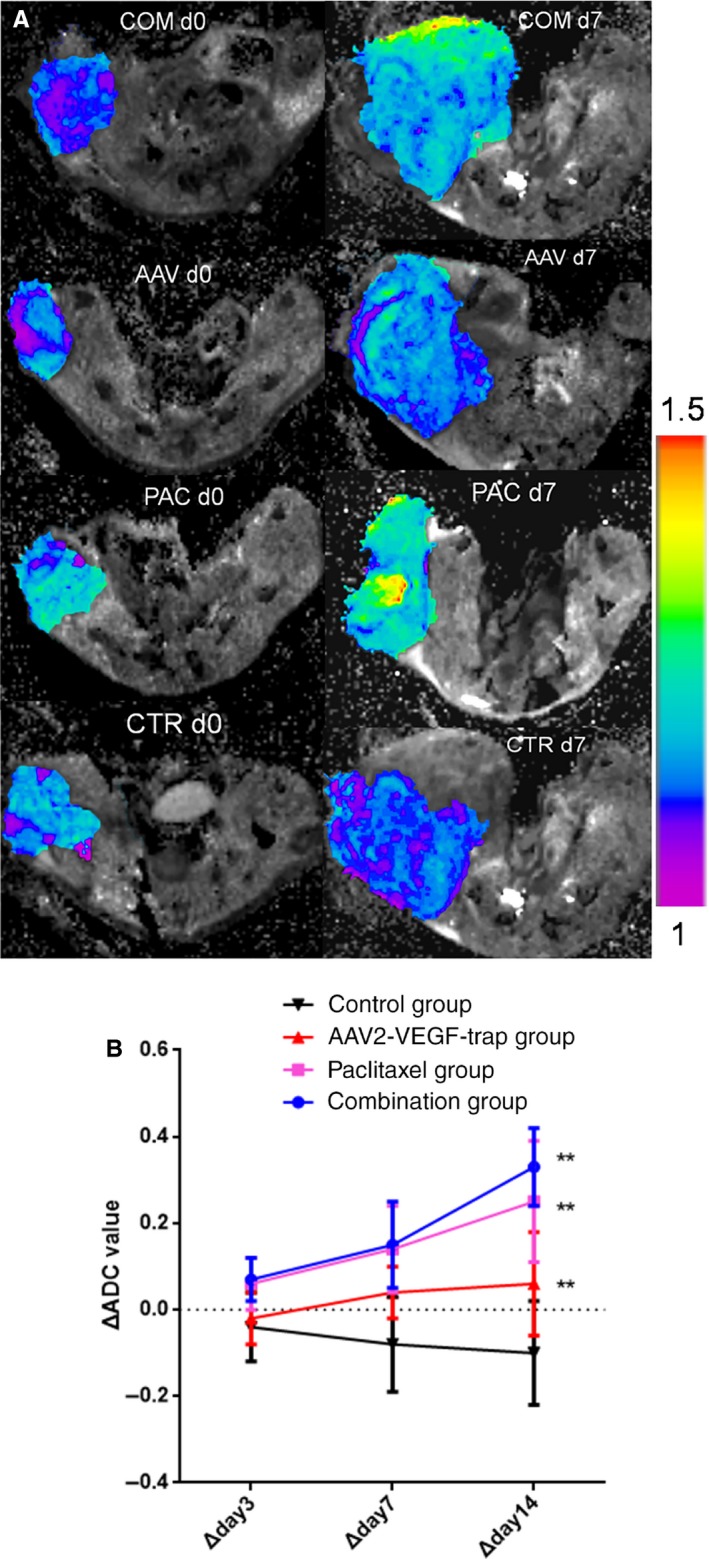
Apparent diffusion coefficient (ADC) maps and ADC/ΔADC values were obtained by MR‐DWI. MR‐DWI was performed on each nude mouse before treatment (day0) and on the 3rd, 7th, and 14th days after treatment followed by measurement of ADC value and ΔADC. A, ADC maps obtained by diffusion‐weighted MRI through the most uniform cross‐section of signal strength of tumor acquired at day 0 and day 7 of treatment. The purple color indicates low ADC value. B, ΔADC values change with time in each group. Abbreviations: COM, AAV2‐VEGF‐Trap combined with paclitaxel group; PAC, paclitaxel group; AAV, AAV2‐VEGF‐Trap group; CTR, control group; d0, day0; d7, day7

We employed DW‐MRI to evaluate the efficacy of neoadjuvant therapy for TNBC. The ADC values (mean ± SD) of each group were recorded before treatment (day 0) and on the 3rd, 7th, and 14th day after treatment. No significant difference was found in baseline ADC values among treatment groups and the control group (Table 1). On the 7th day after administration, the ADC value of the paclitaxel group was significantly different from that of the control group (Table 1, *P* = 0.02). On the 14th day after administration, the ADC value of the AAV2‐VEGF‐Trap combined with paclitaxel group was significantly different from that of the control group (Table 1, *P* < 0.0001). However, no significant difference in ADC values was noted between the AAV2‐VEGF‐Trap and control groups on the 3rd, 7th, and 14th day after treatment (Table 1). The ADC maps revealed that the diffusion signal intensity of the combination group and paclitaxel group was enhanced, whereas the diffusion signal of the control group was reduced (Figure 5A).

**Table 1:** ADC values (mean ± SD) (×10^‐3^ mm^2^/s) of tumors in each group were recorded before treatment (day0) and on the 3rd, 7th, and 14th day after treatment

Group	Simple size	day0	*P* value (day0)	day3	*P* value (day3)	day7	*P* value (day7)	day14	*P* value (day14)
AAV+PAC	7	1.02 ± 0.14	0.24	1.08 ± 0.11	0.90	1.17 ± 0.14	0.08	1.35 ± 0.08	<0.0001
PAC	7	1.11 ± 0.22	0.90	1.18 ± 0.22	0.38	1.26 ± 0.19	0.02	1.36 ± 0.14	0.0001
AAV	10	1.02 ± 0.11	0.16	1.00 ± 0.12	0.24	1.06 ± 0.22	0.84	1.09 ± 0.13	0.21
Control	9	1.13 ± 0.20	‐	1.09 ± 0.19	‐	1.05 ± 0.12	‐	1.01 ± 0.12	‐

*P*‐value was obtained by comparing the treatment group with the control group at each time point.

AAV+PAC, AAV2‐VEGF‐Trap combined with paclitaxel group; ADC, apparent diffusion coefficient; PAC, paclitaxel group; AAV, AAV2‐VEGF‐Trap group.

The rate of change in ADC values (ΔADC) was calculated on the 3rd, 7th, and 14th day after treatment for the four groups (Table 2), and the changing tendency was analyzed (Figure 5B). Over time, the ΔADC values of treatment groups increased to different degrees, whereas that of the control group decreased gradually (Figure 5B). On the 3rd day after administration, the ΔADC values of the paclitaxel group and AAV2‐VEGF‐Trap combined with paclitaxel group were significantly different compared with the control group, and this difference is more pronounced after the 7th and 14th day of administration. On the 14th day after administration, the ΔADC value of theAAV2‐VEGF‐Trap group was significantly different from that of the control group (Table 2).

**Table 2:** The ΔADC value of tumors in each group was recorded on the 3rd, 7th, and 14th day after treatment

Group	simple size	Δday3	*P* (Δday3)	Δday7	*P* (Δday7)	Δday14	*P* (Δday14)
AAV+PAC	7	0.07 ± 0.07	0.005	0.16 ± 0.13	0.001	0.34 ± 0.16	<0.0001
PAC	7	0.06 ± 0.06	0.004	0.14 ± 0.12	0.001	0.25 ± 0.17	0.0001
AAV	10	−(0.02 ± 0.06)	0.62	0.04 ± 0.19	0.15	0.07 ± 0.12	0.005
Control	9	−(0.03 ± 0.05)	‐	−(0.06 ± 0.09)	‐	−(0.09 ± 0.09)	‐

ΔADC = (average ADC value of each time point after treatment‐average ADC value before treatment)/(average ADC value before treatment).

*P*‐value was obtained by comparing the treatment group with the control group at each time point.

AAV+PAC, AAV2‐VEGF‐Trap combined with paclitaxel group; ADC, apparent diffusion coefficient; PAC, paclitaxel group; AAV, AAV2‐VEGF‐Trapgroup.

**Figure 6:**
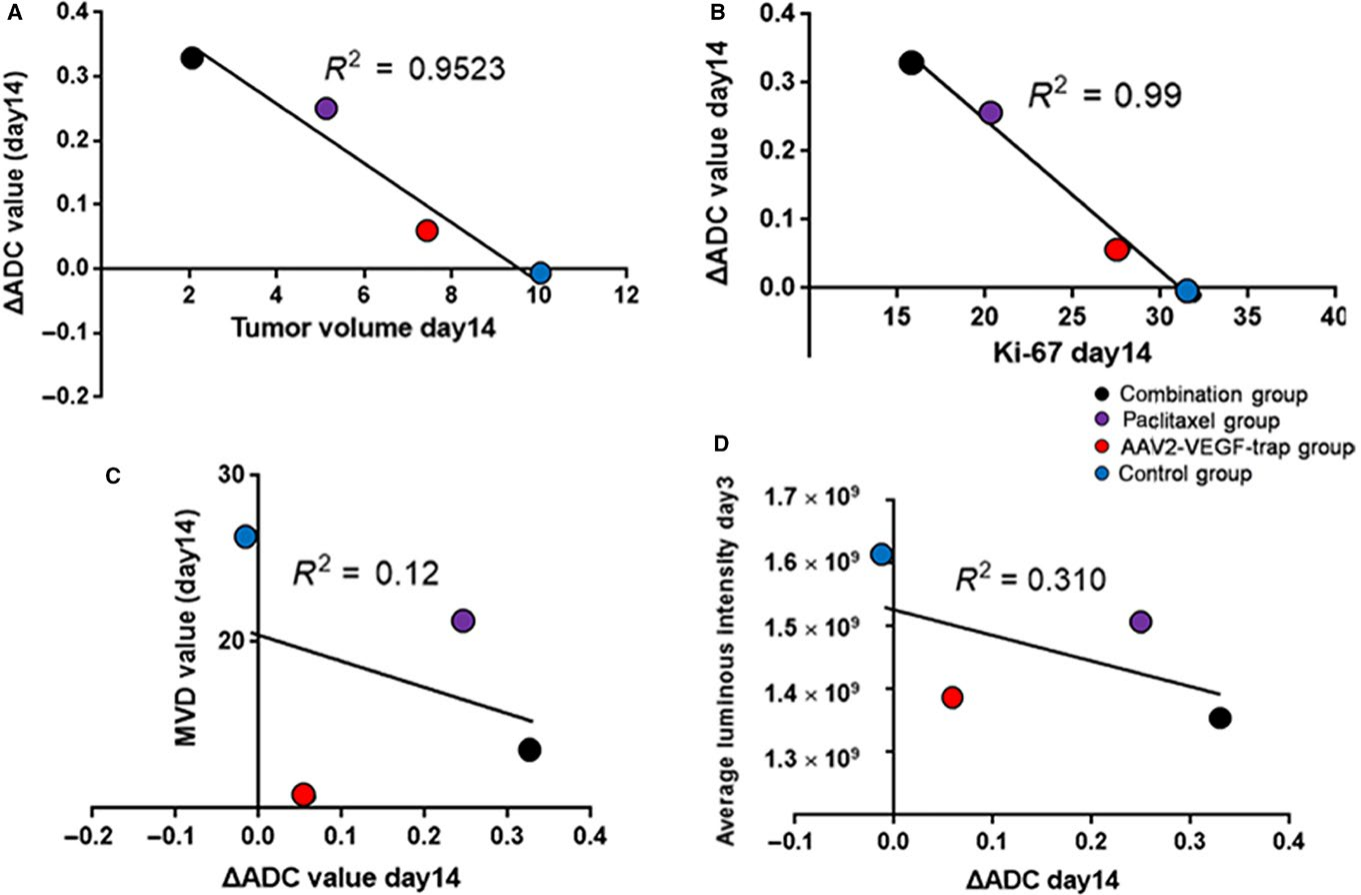
Correlation analysis between endpoint ΔADC values with each evaluating index. A, Correlation analysis between ΔADC values with tumor volume after 14 d of treatment. B, Correlation analysis between ΔADC values with Ki‐67 index after 14 d of treatment. C, Correlation analysis between ΔADC values with MVD after 14 d of treatment. D, Correlation analysis between ΔADC values after 14 d of treatment with average luminous intensity after 3 d of treatment

The correlation among ΔADC, tumor volume, MVD, and Ki‐67 was analyzed. ΔADC was negatively correlated with tumor volume and Ki‐67 (*R* ^2 ^= 0.95, Figure 6A; *R* ^2 ^= 0.99, Figure 6B). Notably, ΔADC value exhibits no obvious correlation with MVD and average luminous intensity (*R* ^2 ^= 0.12 Figure 6C; *R* ^2 ^= 0.31, Figure 6D).

## 4 DISCUSSION

This research demonstrated the anti‐angiogenesis effect of AAV2‐VEGF‐Trap in TNBC (Figure 2A,B). AAV2‐VEGF‐Trap inhibited the growth of TNBC, but it was less efficacious than paclitaxel. AAV2‐VEGF‐Trap significantly enhanced the antitumor activity of paclitaxel in our TNBC animal model (Figure 1). This work provides strong preclinical evidence for the clinical use of AAV2‐VEGF‐Trap combined with paclitaxel for TNBC neoadjuvant/adjuvant therapy.

We assessed the anti‐angiogenesis effect of AAV2‐VEGF‐Trap using MVD andin vivo fluorescence imaging. The MVD of the AAV2‐VEGF‐Trap group and AAV2‐VEGF‐Trap combined with the paclitaxel group were significantly lower than that of control group and paclitaxel group (Figure 2A,B), indicating that AAV2‐VEGF‐Trap can effectively inhibit tumor neovascularization in TNBC. MVD is a validated indicator for accessing the efficacy of anti‐angiogenic agents. MVD was obtained by IHC staining of CD31 on tumor biopsies. In animal experiments, it is often necessary to sacrifice animals in order to obtain tumor tissue, which is not conducive to longitudinal observation of drug efficacy. In the clinic, repeated biopsy is unethical and unrealistic. Therefore, a noninvasive biomarker is needed as a surrogate for MVD. In this study, we use in vivo fluorescence imaging coupled with the optical imaging probe AngioSense680 EX to assess the anti‐angiogenesis effect. The average luminescence intensity of the AAV2‐VEGF‐Trap group and the AAV2‐VEGF‐Trap combined with paclitaxel group was significantly lower than that of paclitaxel group and the control group; the mean luminescence intensity of the AAV2‐VEGF‐Trap group was not significantly different from that of the AAV2‐VEGF‐Trap combined with paclitaxel group (Figure 4B). The result was consistent with the MVD results. Moreover, the average luminescence intensity exhibits a good correlation with MVD (*R* ^2 ^= 0.94, Figure 4C). Therefore, we inferred that the average luminescence intensity obtained by in vivo fluorescence imaging can replace MVD as a noninvasive biomarker for evaluating the efficacy of anti‐angiogenic drugs. However, in vivo fluorescence imaging is used for research and preclinical studies .^14^ Moreover, given the specificity of probe, this method cannot evaluate the efficacy of other non‐anti‐angiogenic drugs.

Response Evaluation Criteria in Solid Tumors (RECIST)^26^ is a commonly accepted standard for assessing tumor response to therapy by measuring tumor size. The reduction in tumor volume occurs late and cannot reflect treatment‐induced physiological changes .^27^ As a noninvasive method to display information about the movement of water molecules in vivo, ADC value was recognized as a biomarker of response to cancer therapy by the International Congress of Magnetic Resonance (ISMRM) in 2008.^16^ Several previous studies have demonstrated increases in ADC values after cytotoxicity treatment .^19^, ^20^ ADC values are affected by many factors. In addition to cell density and cell membrane permeability, magnetic susceptibility, tissue component, temperature, blood flow, and perfusion also affect the ADC values.^19^ On one hand, tumor necrosis or apoptosis after treatment leads to a decrease in tumor cell density and an increase in ADC value; on the other hand, inhibition of neovascularization after anti‐angiogenesis treatment leads to a decrease in blood perfusion, resulting in a decrease in ADC value. Therefore, how the ADC value changes after effective anti‐angiogenesis therapy remains controversial. Using a higher b‐value, the ADC value is less affected by perfusion.^28^ In the present study, *a* *b*‐value of 1000 s/mm^2^ that was sufficient to retain image quality and avoided the influence of perfusion. However, this study confirmed that the ADC value of the AAV2‐VEGF‐Trap group was not significantly different from that of the control group on 3, 7, and 14 days after administration (Table 1). Therefore, the ADC value is not recommended to monitor the efficacy of anti‐angiogenic drugs. The ΔADC value is the increase/decrease rate of the ADC value at a certain time point and is more sensitive than the ADC value to evaluate drug efficacy.^29^ The present study indicated that the ΔADC value exhibits great potential in the evaluation of the response of TNBC to neoadjuvant therapy. Compared with the control group, the ΔADC value of the combination group and paclitaxel group was significantly increased after 3 days of administration. The ΔADC value of the AAV2‐VEGF‐Trap group was significantly increased after 14 days of administration (Table 2). Moreover, the ΔADC value has a strong negative correlation with tumor volume and Ki‐67 index (*R* ^2 ^= 0.95, Figure 6A; *R* ^2 ^= 0.99, Figure 6B, respectively). The other study also reported the same correlation for intracranial tumor patients.^30^ Moreover, no significant correlation was noted between the ΔADC value and MVD as well as the average optical intensity (*R* ^2 ^= 0.12, Figure 6C; *R* ^2 ^= 0.31, Figure 6D, respectively). These results demonstrate that the ΔADC value is not affected by blood perfusion and micro vessel density. This study confirmed that ΔADC can dynamically and longitudinally monitor the efficacy of TNBC neoadjuvant therapy independent of the drug's mechanism of action. Therefore, ΔADC can replace tumor volume as a biological indicator of the response to neoadjuvant therapy.

Several potential limitations of this study should be considered. Due to the small sample size of each group, false negatives may occur during statistical analysis. Another limitation of this experiment is the lack of adequate participants for follow‐up. This study used a mouse model, and further clinical retrospective or prospective studies are needed.

## 5 CONCLUSION

A single intravenous dose of AAV2‐VEGF‐Trap inhibits angiogenesis and growth in a TNBC animal model and exhibits a synergistic effect with paclitaxel. The average luminescence intensity obtained by in vivo fluorescence imaging can replace MVD as a biomarker to assess the efficacy of anti‐angiogenic agents. DW‐MRI can longitudinally monitor the neoadjuvant efficacy of TNBC.

## ETHICAL APPROVAL

All applicable international, national, and/or institutional guidelines for the care and use of animals were followed.

## CONFLICT OF INTEREST

All authors declare that they have no conflict of interests.
